# Aided Recognition and Training of Music Features Based on the Internet of Things and Artificial Intelligence

**DOI:** 10.1155/2022/3733818

**Published:** 2022-03-11

**Authors:** Xidan Zhang

**Affiliations:** Teacher College, Columbia University, New York 10027, NY, USA

## Abstract

With the development of the Internet of Things, many industries have been on the train of the information age, and digital audio technology is also constantly developing. Music retrieval has gradually become a research hotspot in the music industry. Among them, the auxiliary recognition of music characteristics is also a particularly important Task. Music retrieval is mainly to manually extract music signals, but now the music signal extraction technology has encountered a bottleneck. The article uses Internet and artificial intelligence technology to design an SNN music feature recognition model to identify and classify music features. The research results of the article show (1) statistic graphs of the main melody and accompanying melody of different music. The absolute value of the main melody and accompanying melody mainly fluctuates in the range of 0–7, and the proportion of the main melody can reach 36%. The accompanying melody can reach 17%. After the absolute value of the interval reaches 13, the interval ratio of the main melody and the accompanying melody tends to be stable, maintaining between 0.6 and 0.9, and the melody interval ratio value completely coincides; the main melody in the interval variable is *X*. (1) The relative difference value in the interval of −*X*(16) fluctuates greatly. After the absolute value of the interval reaches 17, the interval ratio of the main melody and the accompanying melody tends to be stable, maintaining between 0.01 and 0.04 and the main melody. The value of the difference is always higher than the accompanying melody. (2) When the number of feature maps is 24*∗*5, the recognition result is the most accurate, MAP recognition result can reach 78.8, and the recognition result of precision@ is 79.2; when the feature map size is 5*∗*5, the recognition result is the most accurate, MAP recognition result can reach 78.9, the recognition result of precision@ is 79.2, and the recognition result of HAM2 (%) is 78.6. The detection accuracy of the SNN music recognition model proposed in the article is the highest. When the number of bits is 64, the detection accuracy of the SNN detection model is 59.2%, and the detection accuracy of the improved SNN music recognition model is 79.3%, which is better than the detection rate of ITQ music recognition model of 17.9%, which is 61.4% higher. The experimental data further shows that the detection efficiency of the ITQ music recognition model is the highest. (3) The SNN music recognition model proposed in the article has the highest detection accuracy, regardless of whether it is in a noisy or no-noise music environment, with an accuracy rate of 97.97% and a detection accuracy value of 0.88, which is 5 types of music. The highest one among the recognition models, the ITQ music recognition model, has the lowest detection accuracy, with a detection accuracy of 67.47% in the absence of noise and a detection accuracy of 70.23% in the presence of noise. Although there is a certain noise removal technology, it can suppress noise interference to a certain extent, but cannot accurately describe music information, and the detection accuracy rate is also low.

## 1. Introduction

Because the network has the advantages of fast information dissemination, easy use, and sufficient network resources, it is widely used in human work and study life. At present, with the rapid development of popular music in our country, music is everywhere, and the music wave has also affected us. When faced with a wide variety of music types, users will inevitably feel at a loss. Users need to spend a lot of time choosing the type of music they are interested in. This method is not only a waste of time, but also very inefficient. Based on the above background, it is inevitable to design an intelligent auxiliary model of music characteristics. Literature [1] studied the ability of using self-organizing neural mapping as a music style classifier for music fragments. The article cuts the music melody into many segments of equal length, then analyzes the music melody and rhythm, and presents the analyzed data to SOM. Document [2] discloses a system and method for implementing a simple and fast real-time single note recognition algorithm based on fuzzy pattern matching. The system can accept the music rhythm and notes during the performance, and then compared with the correct music rhythm, you can know whether the music rhythm during the performance is standard. Literature [3] proposed a new method for automatic music genre recognition in the visual domain using two texture descriptors. Literature [4] introduces the use of a dynamic set of classifier selection schemes and creates a classifier pool to perform automatic music genre classification. The working principle of the classifier is the principle of support vector machine, which can extract effective information from the spectrum image of music. The research results of the article show that the accuracy of music extraction can reach 83%. Literature [5] introduced optical music recognition technology and proposed a method for computer to automatically recognize music scores. The system can scan the printed images of music scores to extract effective information and then automatically generate audio files to provide users with listening functions. Literature [6] proposed a statistical method to deal with the task of handwritten music recognition in early notation. This method of processing music is different from the traditional method in that it directly recognizes the music signal without dividing the music signal into many paragraphs. Literature [7] investigated various aspects of automatic emotion recognition in music. Music is also a good way to express emotions. Different classifications and timbres in music will interpret different musical effects. This article explores the extensive research on music emotion recognition. Literature [8] studied the utility of the most advanced pretraining deep audio embedding method used in the task of music emotion recognition. Literature [9] proposed a music emotion recognition method based on adaptive aggregation regression model. Emotion recognition of music is an important task to evaluate the influence of music on the emotions of listeners. The article proposes an emotion estimation model, which uses the variance obtained by Gaussian process regression to measure the confidence of the estimation results of each regression model. Literature [10] proposed a new method of using template matching and pixel pattern features in computer games. The general music model does not have much to do with the change of the font, but the beats and notes of some notes do not maintain the original shape of the music signal. The model proposed in the article can be applied to these music symbols. Literature [11] proposed a method to solve the problem of multidimensional music emotion recognition, combining standard and melody audio features. Literature [12] studied the reduction of the number of training examples in music genre recognition. The article studies the impact of the reduction of training real numbers on the detection results in the process of music style recognition. The experimental results show that although the number of experiments is greatly reduced during the detection process, it can still maintain a high classification performance in many cases. Literature [13] presents a method to parse solo performances into individual note components and use support vector machines to adjust the back-end classifier. In order to realize the generalization of instrument recognition to ready-made, commercial solo music, [14] proposed a method of musical instrument recognition in chord recording. Literature [15] proposed a method for analyzing and recognizing music speech signals based on speech feature extraction. The method is to extract effective music information from the music signal and then reorganize the music signal to a certain extent, so as to achieve the function of noise reduction. The results of the experiment show that the reorganized music signal has good noise reduction compared with the original music signal ability.

## 2. Research on Auxiliary Recognition of Music Features

### 2.1. Overall Structure of Music Feature Recognition

**Figure 1:**
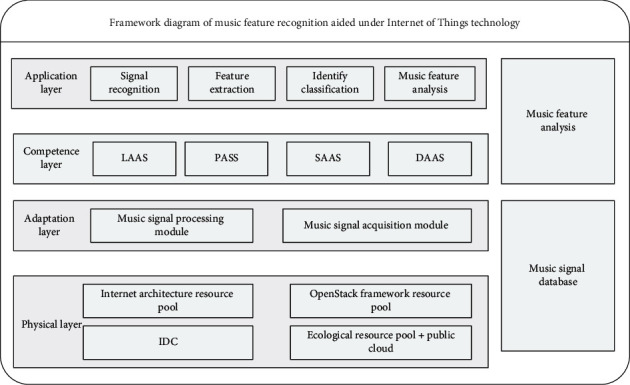
Framework diagram of music feature recognition.

The music feature recognition system based on the Internet of Things technology is mainly composed of a physical perception layer, a capability layer, an adaptation layer, and a system application layer. The overall structure of the system is shown in Figure 1.

### 2.2. Design of Music Collection Module

To identify the music signal, it is necessary to collect the music signal first. The music collection module is composed of two parts, namely, the collection submodule and the encoding module. The music collection submodule is composed of sound sensors installed in different positions and is responsible for collecting the original music signal [16]. The sound sensor has a built-in capacitive electret microphone that is sensitive to sound, which is converted by an A/D converter and transmitted to the voice coding submodule [17]. The voice coding submodule is mainly responsible for the high-fidelity and lossless compression of the original music signal, converts the music signal into transmittable data information, and then transmits it to the music signal processing module.

### 2.3. Music Signal Module Processing Design

**Figure 2:**
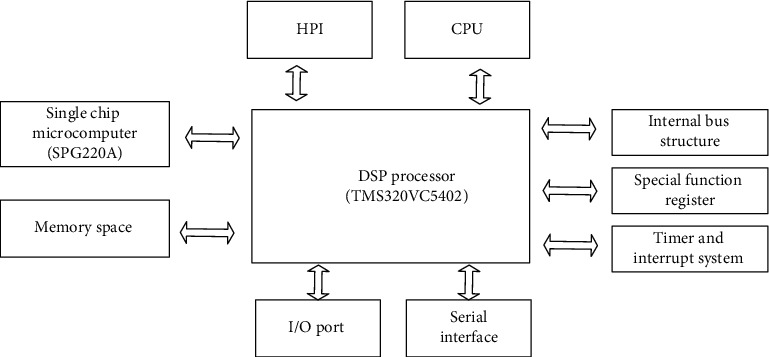
Functional structure diagram.

The music signal processing module is designed by a DSP processor [18]. The module uses a fixed DS chip suitable for voice signal processing. The DSP chip has low power consumption and fast running speed. It carries 2 MCBSPS, can be connected to CODEC for voice input, and has an 8-bit enhanced host parallel port to communicate with the host. Establish a communication connection, including 4 KB ROM and 16 KB DARAM. Its structure is shown in Figure 2:

## 3. Music Feature Assisted Recognition and Training

### 3.1. Extraction of Basic Music Features

Pitch, time value, and tone intensity are the most basic elements of music characteristics. The formula for the pitch level of music is defined as

(1) $$\begin{array}{c} \bar{p}=\frac{\sum_{i=1}^{n}pi}{n}. \end{array}$$

*pi* represents the pitch of the *i* note, and *n* represents the number of notes in the music.

Treble changes:

(2) $$\begin{array}{c} \text{Var}\_p=\frac{\sum_{i=1}^{N-1}{\text{Bar}}_{i+1}-{\text{Bar}}_{i}}{N\bar{P}}. \end{array}$$

The pitch mean square error can be used to express the pitch change:

(3) $$\begin{array}{c} \text{Var}\_P=\sqrt{\frac{1}{n}\overset{n}{\underset{i=1}{\sum }}{\left(P_{i}-\frac{\sum_{i=1}^{n}P_{i}}{n}\right)}^{2}}. \end{array}$$

The range describes the breadth of the pitch of the music:

(4) $$\begin{array}{c} \text{range}=\text{Max}\left(P_{1},P_{2},\ldots ,P_{n}\right)-\text{Min}\left(P_{1},P_{2},\ldots ,P_{n}\right). \end{array}$$

Time value:

(5) $$\begin{array}{c} \text{duration}=\text{end time}-\text{start time}. \end{array}$$

#### 3.1.1. Tone and Music Feature Extraction

The frequency spectrum distribution of music signals and the emotions expressed by timbre perception are shown in Table 1 [19].

The formula for extracting music strength is

(6) $$\begin{array}{c} \text{Dyn}=\frac{1}{n}\overset{n}{\underset{i=1}{\sum }}I_{t},\\ \text{Var}\_\text{Dyn}=\sqrt{\frac{1}{m}\overset{m}{\underset{i=1}{\sum }}\left(I_{i}-\frac{\sum_{i=1}^{m}I_{i}}{m}\right)}. \end{array}$$

The degree of musical intensity change can also be expressed as

(7) $$\begin{array}{c} \text{Var}\_\text{Dyn}=\frac{\overset{N-1}{\underset{i=1}{\sum }}D_{t+1}-D_{t}}{N\cdot \text{Dyn}}. \end{array}$$

#### 3.1.2. Melody Direction Recognition

The expression formula of music melody is

(8) $$\begin{array}{c} \text{Mel}=\overset{n-1}{\underset{i=1}{\sum }}\frac{\left(P_{i+1}-P_{i}\right)\cdot D_{i}}{D-D_{n}}. \end{array}$$

*D* represents the total length of all notes; *D*_*i*_ represents the length of the *i*-th note [20].

The melody direction can also be expressed as

(9) $$\begin{array}{c} \text{Mel}=\overset{n-1}{\underset{i=1}{\sum }}\frac{P_{i+1}-P_{i}}{D_{i}}. \end{array}$$

The expression formula of pronunciation point density is

(10) $$\begin{array}{c} \text{density}=\frac{n}{D}. \end{array}$$

The change intensity of the rhythm is

(11) $$\begin{array}{c} \text{Rhy}=\overset{n-1}{\underset{i=1}{\sum }}\left|\frac{I_{i+1}-I_{i}}{D_{i}}\right|. \end{array}$$

Music mutation degree:

(12) $$\begin{array}{c} \text{mutation}=\text{Max}\left(\frac{\left|\text{BarCapacity}\left(i\right)-\text{BarCapacity}\left(i-1\right)\right|}{\text{Max}\left(\text{BarCapacity}\left(i\right)\right)}\right). \end{array}$$

The expression of BarCapacity is [21]

(13) $$\begin{array}{c} \text{BarCapacity}=\overset{n}{\underset{i=0}{\sum }}\frac{K\left(f_{0}\right)\cdot D_{i}\cdot I_{i}}{j},\\ K\left(f_{0}\right)=\left\{\begin{array}{c} \frac{90}{120-\left(30f_{0}/500\right)},\;\text{when }20\text{ Hz}≺f_{0}≺500\text{ Hz}\\ 1,\;\text{when }500\text{ Hz}≺f_{0}≺1000\text{ Hz,}\\ \frac{90}{90-\left(10\times \left(f_{0}-1000\right)/4000\right)},\;\text{when }1000\text{ Hz}≺f_{0}≺5000\text{ Hz,} \end{array}\right.\\ j=\frac{\sum_{i=0}^{n}{\text{Velocity}}_{i}}{120}. \end{array}$$

### 3.2. Musical Inference Rules

Sudden changes in treble or tone stability appear in the sequence variance. In order to measure these change points, first express the music as the following time sequence:

(14) $$\begin{array}{c} Y_{k}=\mu +\varepsilon_{k},\;k=0,1,2,3,\cdots ,T. \end{array}$$

Among them, *μ* represents the unknown constant mean value of time series *Y*_*k*_, and *σ*^2^ represents the unknown constant variance of time series *Y*_*k*_ (and *ε*_*k*_).

Get the iterative residual sequence:

(15) $$\begin{array}{c} a_{k}=\frac{Y_{k}-\left(\sum_{i=0}^{k-1}\left(Y_{i}/k\right)\right)}{\sqrt{\left(k+1/k\right)S_{y}^{2}}},\;k=0,1,2,3,\cdots ,T, \end{array}$$

and make

(16) $$\begin{array}{c} C_{k}=\overset{k}{\underset{i=1}{\sum }}a_{i}^{2}\;k=0,1,2,3,\cdots ,T. \end{array}$$

Get statistics:

(17) $$\begin{array}{c} W_{k}=\frac{C_{k}}{C_{T}},\;k=0,1,2,3,\cdots ,T. \end{array}$$

After centralized processing,

(18) $$\begin{array}{c} D_{k}=\frac{C_{k}}{C_{T}}-\frac{k}{T},\;k=0,1,2,3,\cdots ,T,D_{0}=D_{T}=0. \end{array}$$

### 3.3. Music Separation Algorithm

According to the difference between the impact sound and the harmonic sound in the frequency spectrum, we can separate the original spectrum *W*_*fJ*_ into the impact spectrum *P*_*fJ*_ and the harmonic spectrum, which is

(19) $$\begin{array}{c} W_{fJ}=P_{fJ}+H_{fJ}. \end{array}$$

The separation of impact sound and harmonic sound:

(20) $$\begin{array}{c} Q\left(H^{t},P^{t},U^{t},V^{t}\right)=\frac{1}{\sigma_{H}^{2}}\underset{fJ}{\sum }\left\{{\left(H_{f,t-1}^{t}-U_{f,t}^{t}\right)}^{2}-{\left(H_{f,t}^{t}-U_{f,t}^{t}\right)}^{2}\right\}+\frac{1}{\sigma_{p}^{2}}\underset{fJ}{\sum }\left\{{\left(P_{f,t-1}^{t}-V_{f,t}^{t}\right)}^{2}-{\left\{P_{f,t}^{t}-V_{f,t}^{t}\right\}}^{2}\right\}. \end{array}$$

Minimum:

(21) $$\begin{array}{c} H_{f,J}^{t+1}=H_{fJ}^{t}+\Delta^{t},\\ P_{f,J}^{t+1}=P_{fJ}^{t}+\Delta^{t}, \end{array}$$

in

(22) $$\begin{array}{c} \Delta^{t}=\frac{\alpha }{4}\left(H_{f\;J-1}^{t}-2H_{f\;t+1}^{t}+H_{f\;t+1}^{t}\right)-\frac{1-\alpha }{4}\left(P_{f\;J-1}^{t}-2P_{f\;t+1}^{t}+P_{f\;t+1}^{t}\right),\\ \alpha =\frac{\sigma_{\gamma }^{2}}{\sigma_{H}^{2}+\sigma_{\gamma }^{2}}. \end{array}$$

## 4. Simulation Experiment

### 4.1. Music Feature Recognition

#### 4.1.1. Algorithm Definition

Algorithm definition is as shown in Table 2.

#### 4.1.2. Experimental Data and Research

**Figure 3:**
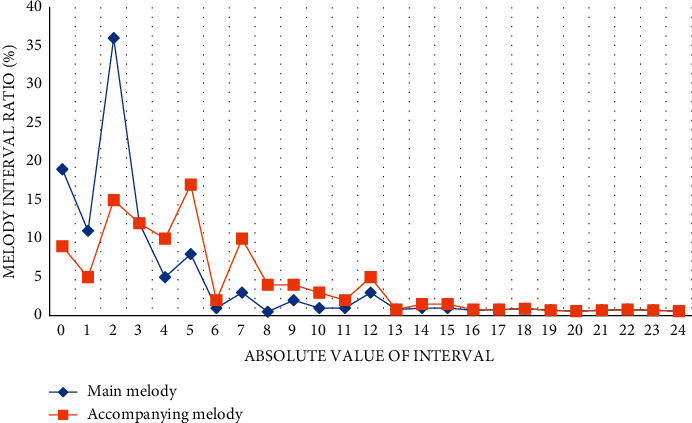
Interval statistics of different audio tracks.

The article uses the Internet of Things and human intelligence technology to design an SNN music feature assisted recognition model. In order to detect the recognition efficiency of the SNN music feature assisted recognition model, the experiment selected more than 50 pieces of multiple types of music for music feature recognition and counted them separately: the main melody and accompanying melody curves of different music. The main melody lines of different types of music are different, and the main characteristics of music melody are linearity and fluidity. The abscissa of the experimental statistics graph represents the absolute value of the interval, and the ordinate represents the percentage of the absolute value of the interval. The specific experimental results are shown in Figure 3.

From the data in Figure 3, we can conclude that the absolute value of the interval between the main melody and the accompanying melody mainly fluctuates in the range of 0–7. In the interval line chart of the main melody, the second degree accounted for the highest proportion of the interval melody. Reaching 36%, in the interval line chart accompanying the melody, 5 degrees accounted for the highest proportion of interval melody, up to 17%. After the absolute value of the interval reaches 13, the interval ratio of the main melody and the accompanying melody tends to be stable, maintaining between 0.6 and 0.9, and the melody interval ratio values completely coincide.

**Figure 4:**
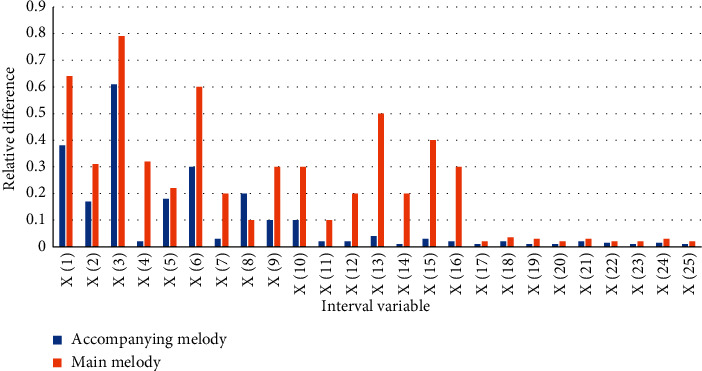
Relative difference distribution of interval statistics.

According to the experimental data in Figure 4, we can conclude that the relative difference of the main melody within the interval of *X*(1)–*X*(16) fluctuates greatly. When the interval variable is *X*(3), the relative difference is the largest. The maximum can reach 0.79. The relative difference value of the accompanying melody in the interval variable *X*(1)–*X*(10) fluctuates greatly. When the interval variable is *X*(3), the relative difference value is the largest, and the maximum can reach 0.61. After the absolute value of the interval reaches 17, the interval ratio of the main melody and the accompanying melody tends to be stable, maintaining between 0.01 and 0.04, and the difference between the main melody and the accompanying melody is always higher than that of the accompanying melody.

### 4.2. Comparative Experiment and Analysis

#### 4.2.1. The Influence Experiment of Feature Map

Based on the same recognition results of different features, it can directly reflect the recognition accuracy of different models and experimentally study the influence of the number and size of feature maps on the detection results. In the different feature map number recognition experiment, different distributions of convolutional layers were selected, and the distribution size was from 8 to 64. In the different feature map size recognition results experiment, 11 feature maps of different sizes were selected. The experimental data is shown in Tables 3 and 4.

**Figure 5:**
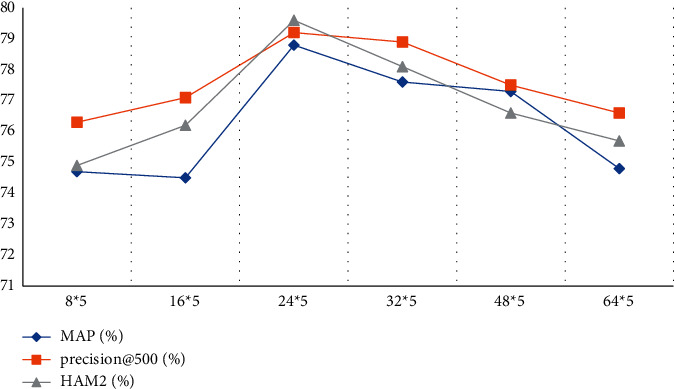
Statistics of recognition results.

According to the data in Table 3 and Figure 5, we can conclude that when the number of feature maps is 24*∗*5, the recognition result is the most accurate, the MAP recognition result can reach 78.8, the recognition result of precision@ is 79.2, and the recognition result of HAM2 (%) is 79.6. When the number of feature maps is 8*∗*5, the accuracy of the recognition result is the lowest. The recognition rate of MAP is 74.7, the recognition result of precision@ is 76.3, and the recognition result of HAM2 (%) is 74.9. In general, the detection accuracy of 6 different numbers of feature maps generally maintains above 74%.

**Figure 6:**
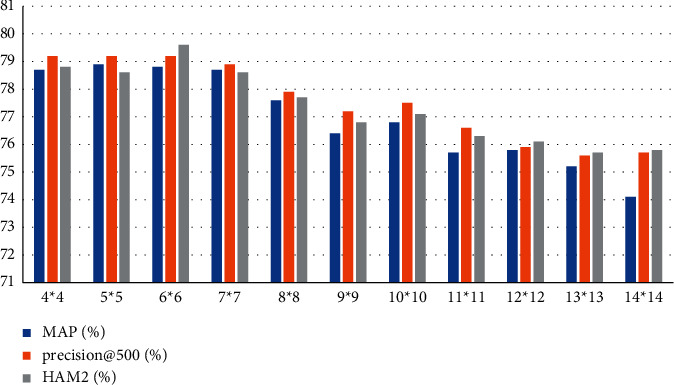
Statistics of recognition results.

According to the data in Table 4 and Figure 6, we can conclude that when the feature map size is 5*∗*5, the recognition result is the most accurate. The MAP recognition result can reach 78.9, the recognition result of precision@ is 79.2, and the recognition result of HAM2 (%) is 78.5. When the feature map size is 78.6 and the feature map size is 14*∗*14, the recognition accuracy is the lowest. The recognition accuracy of MAP is 74.1, the recognition result of precision@ is 75.7, and the recognition result of HAM2 (%) is 75.8. In general, the detection accuracy of 11 different sizes of feature maps generally maintains above 74%.

#### 4.2.2. Comparison with Other Methods

In order to test the performance of the music recognition model, the experiment improved the SNN music recognition model proposed in the article and compared it with the detection performance of the other three models. The experiment chose 5 different types of bit numbers. The number of bits is a unit, and the same as the sampling accuracy, the higher the baud rate or bit rate is, the more detailed the light changes of the music can be reflected. Observe the detection accuracy rates of 5 different models under different types of bits. The specific experimental data are shown in Table 5.

**Figure 7:**
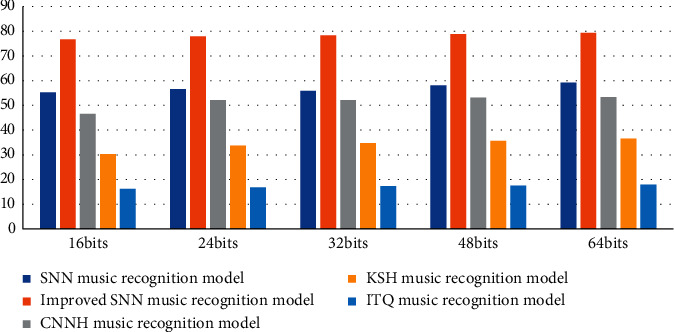
Average mean precision statistics.

According to the data in Table 5 and Figure 7, we can conclude that the detection accuracy of the SNN music recognition model proposed in the article is the highest among 5 different music recognition models. When the number of bits is 64, the SNN detection accuracy rate of the improved SNN music recognition model is 59.2%, and the detection accuracy rate of the improved SNN music recognition model is 79.3%, which is 61.4% higher than the 17.9% detection rate of the ITQ music recognition model. The experimental data further shows that the ITQ music recognition model has the highest detection efficiency, which greatly promotes the efficiency of music feature auxiliary recognition.

### 4.3. Test Model Performance Comparison Test

#### 4.3.1. Evaluation Criteria

The evaluation criteria are as shown in Table 6.

#### 4.3.2. Experimental Results and Analysis

In order to test the performance of the SNN music feature-assisted recognition model, we run the model proposed in the article and other music recognition models under noisy and no-noise music conditions, observe the detection accuracy of different models, and verify the detection accuracy of different models. In order to make the experimental results more analytical, we have selected 5 different types of music data. The experiment detects these 5 types of music data with and without noise and observes the experimental results. The music sample data is shown in Table 7, and the specific detection results are shown in Tables 8 and 9.

**Figure 8:**
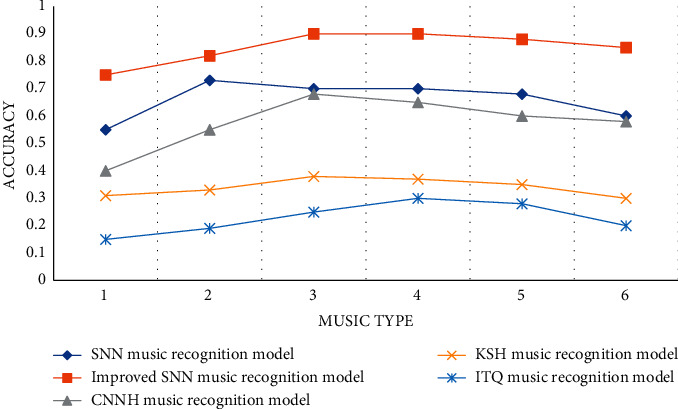
Comparison of uninteresting music classification and detection accuracy.

According to the data in Table 8 and Figure 8, we can conclude that the SNN music recognition model proposed in the article has the highest detection accuracy, with an accuracy rate of 97.97% and a detection accuracy value of 0.88. It is 5 types of music recognition models. The tallest one among them, the ITQ music recognition model, has the lowest detection accuracy rate of 67.47%, and the highest detection accuracy value is 0.3. The CNNH music recognition model and the KSH music recognition model are in the middle of the highest value and the lowest value.

**Figure 9:**
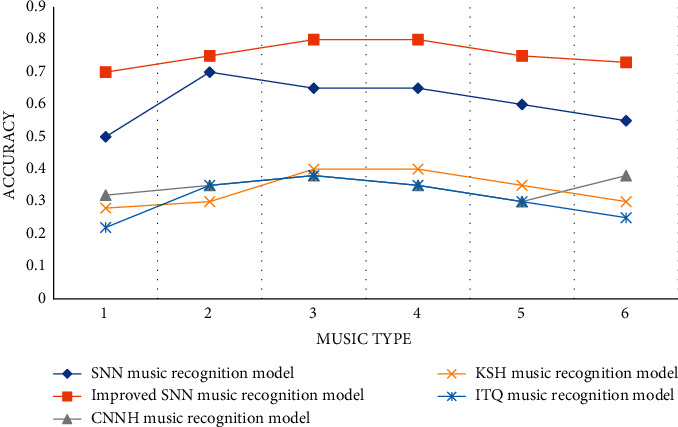
Comparison of dry music classification and detection accuracy.

We can find from Figure 9 that the ITQ music recognition model has the lowest detection accuracy. The detection accuracy rate in the absence of noise is 67.47%, and the detection accuracy in the presence of noise is 70.23%. Although there is a certain noise removal technology, it can suppress noise interference to a certain extent, but cannot accurately describe music information, and the detection accuracy rate is also low. The detection accuracy of the KSH music recognition model is higher than that of the ITQ music recognition model, which can accurately describe the changes of music signals, but there are certain defects in noise processing, and the error rate of music detection is relatively large. The SNN music feature assisted recognition model proposed in the article has the highest detection accuracy among the five models, and there are many types of music detected, which can analyze music signals more comprehensively and systematically, and the accuracy rate is as high as 99.12%, thus greatly improving the efficiency of music detection. It is believed that the detection accuracy will be improved by using feature extraction approaches.

## 5. Conclusion

Today we are in an era of informationization and intelligence. The use of intelligent methods to study music has attracted more and more people's attention. Computer music has also made many achievements and has a very broad market prospect. Using a computer to simulate music signals, this process involves not only computers and music, but also a lot of complex professional knowledge. At present, there are still many problems in the auxiliary recognition of music characteristics in human intelligence; despite the design of the auxiliary model of music characteristics recognition in the article, music signals can be analyzed and identified efficiently, but the way of expressing music needs further research.

## Figures and Tables

**Table 1:** Tone-emotion mapping relationship.

Musical emotion type	Tonal characteristics
Hate class	The tone is sharp, rough, and bright
Depression	The tone is deep, pure, simple, monotonous bass, dim, plain, and hollow
Calm meditation class	The tone is soft, pure, and simple
Desire	The tone is deep, plain, hollow, pure, and simple
Pastoral style	The tone is soft, pure, and simple
Perceptual	The tone is soft, sweet and soft, rich, gorgeous, pleasant, and nasal
Active class	The tone is bright, rich, and gorgeous
Awesome	The tone is bright, sharp, and brilliant

**Table 2:** Algorithm definition table.

	Definition	Publicity
Interval statistics	Statistics of the melody interval are carried out on each track of the music [22]	$\text{Interval}\_{\text{Sta}}_{i}=\left\{\begin{array}{c} \left|{\text{Interval}}_{i}\right|,\left|{\text{Interval}}_{i}\right|<25\\ 25,\left|{\text{Interval}}_{i}\right|\geq 25 \end{array}\right.$
Classification algorithm	According to the different characteristics of the interval distribution, the main track and the accompanying track are distinguished [23]	Rhythm=(*n*/*T*)=(*n*/(Dura_Meter × Dura_Num))

**Table 3:** Recognition results of different number of feature maps.

Quantity	MAP (%)	Precision@500 (%)	HAM2 (%)
8*∗*5	74.7	76.3	74.9
16*∗*5	74.5	77.1	76.2
24*∗*5	78.8	79.2	79.6
32*∗*5	77.6	78.9	78.1
48*∗*5	77.3	77.5	76.6
64*∗*5	74.8	76.6	75.7

**Table 4:** Recognition results of different feature map sizes.

Quantity	MAP (%)	Precision@500 (%)	HAM2 (%)
4*∗*4	78.7	79.2	78.8
5*∗*5	78.9	79.2	78.6
6*∗*6	78.8	79.2	79.6
7*∗*7	78.7	78.9	78.6
8*∗*8	77.6	77.9	77.7
9*∗*9	76.4	77.2	76.8
10*∗*10	76.8	77.5	77.1
11*∗*11	75.7	76.6	76.3
12*∗*12	75.8	75.9	76.1
13*∗*13	75.2	75.6	75.7
14*∗*14	74.1	75.7	75.8

**Table 5:** Average mean precision of different number of bits.

Method	16 bits	24 bits	32 bits	48 bits	64 bits
SNN music recognition model	55.2	56.6	55.8	58.1	59.2
Improved SNN music recognition model	76.7	77.9	78.3	78.9	79.3
CNNH music recognition model	46.5	52.1	52.1	53.2	53.3
KSH music recognition model	30.3	33.7	34.7	35.6	36.5
ITQ music recognition model	16.2	16.9	17.3	17.5	17.9

**Table 6:** Evaluation criteria table.

Index	Metrics	Formula
Accuracy	The accuracy measurement standard refers to the ratio of the number of correct music types to the number of all music types [24]. The larger the index value, the more accurate the recognition result	Precision=(hits_*u*_/recset_*u*_)
Recall rate	The recall rate standard refers to the proportion of the theoretically largest number of hits that recognize musical characteristics [25]. The larger the index value, the more accurate the recognition result	Recall=(hits_*u*_/testset_*u*_)
*F*1 measurement	The *F*1 measurement index can effectively balance the accuracy rate and the recall rate by favoring the smaller value. The larger the index value, the more accurate the recognition result	*F*1=(2 × precision × recall/(precision+recall))

**Table 7:** Music classification and detection object table.

Music type number	Noisy	No noise
1	10	30
2	10	30
3	20	40
4	10	40
5	20	50
6	20	50

**Table 8:** No-noise recognition results.

Model	Accuracy (%)	Accuracy (%)	Recall rate (%)	*F*1 score (%)
SNN music recognition model	95.71	96.83	96.74	95.82
Improved SNN music recognition model	97.97	98.10	97.89	98.00
CNNH music recognition model	80.21	82.31	83.24	84.51
KSH music recognition model	72.14	73.46	90.26	91.32
ITQ music recognition model	67.47	68.24	66.76	67.12

**Table 9:** Noisy recognition results.

Model	Accuracy (%)	Accuracy (%)	Recall rate (%)	*F*1 score (%)
SNN music recognition model	92.12	91.83	91.64	91.28
Improved SNN music recognition model	93.91	94.21	94.74	94.62
CNNH music recognition model	75.32	77.23	76.34	77.21
KSH music recognition model	68.62	69.24	69.12	68.24
ITQ music recognition model	70.23	71.22	74.21	72.45

## Data Availability

The experimental data used to support the findings of this study are available from the corresponding author upon request.
